# Children and young people’s experiences of completing mental health and wellbeing measures for research: learning from two school-based pilot projects

**DOI:** 10.1186/s13034-020-00341-7

**Published:** 2020-09-16

**Authors:** Ola Demkowicz, Emma Ashworth, Rosie Mansfield, Emily Stapley, Helena Miles, Daniel Hayes, Kim Burrell, Anna Moore, Jessica Deighton

**Affiliations:** 1 Manchester Institute of Education, The University of Manchester, Manchester, UK; 2 School of Psychology, Liverpool John Moores University, Liverpool, UK; 3 Evidence Based Practice Unit (EBPU), University College London and the Anna Freud National Centre for Children and Families, London, UK; 4 Common Room Consulting, London, UK

**Keywords:** Mental health outcomes, Wellbeing, Measurement, Child and adolescent mental health, Self report, School surveys, Measure design, Research ethics

## Abstract

**Background:**

In recent years there has been growing interest in child and adolescent mental health and wellbeing, alongside increasing emphasis on schools as a crucial site for research and intervention. This has coincided with an increased use of self-report mental health and wellbeing measures in research with this population, including in school-based research projects. We set out to explore the way that children and young people perceive and experience completing mental health and wellbeing measures, with a specific focus on completion in a school context, in order to inform future measure and research design.

**Methods:**

We conducted semi-structured interviews and focus groups with 133 participants aged 8–16 years following their completion of mental health and wellbeing measures as part of school-based research programmes, using thematic analysis to identify patterns of experience.

**Findings:**

We identified six themes: *Reflecting on emotions during completion; the importance of anonymity; understanding what is going to happen; ease of responding to items; level of demand; and interacting with the measure format.*

**Conclusions:**

Our findings offer greater insight into children and young people’s perceptions and experiences in reporting on their mental health and wellbeing. Such understanding can be used to support more ethical and robust data collection procedures in child and adolescent mental health research, both for data quality and ethical purposes. We offer several practical recommendations for researchers, including facilitating this in a school context.

## Background

The mental health and wellbeing of children and young people (CYP) has become an international priority in recent years [1, 2], and there is growing recognition of the need for research in this area [3–7]. Self-report measures^1^ are often used across this research agenda, given emerging evidence that CYP are able to report and describe their own health experiences [8–10]. Informant discrepancies between reports of CYP and their parents, once seen as attributable to differences in “accuracy”, are now more commonly thought to reflect differences in perspective, with CYP offering valid and important insights into their own health [11–13]. This also reflects an increased emphasis on the voice of CYP in research and policy, with a “no decision about me without me” approach frequently adopted [14–18].

Given growing engagement and involvement of CYP in research, a range of general guidance has become available, offering both practical and ethical methodological advice (e.g., [19–21]). However, as noted by Crane and Broome in a recent review of the literature [22], there are particular aspects and types of research participation that can affect the way that CYP view and cooperate with research procedures. For instance, a focus on health compromising behaviours (e.g., drug use or suicidal ideation) can prompt suspicion around purported confidentiality procedures [23], while trust in researchers may influence level of cooperation in participation [24]. Investigating potentially sensitive topics, such as mental health, entails a range of considerations, given both ethical concerns regarding participants’ wellbeing and data implications relating to the reliability and validity of results [9, 25]. Social desirability, for instance, can be a central issue with both adults and younger participants, necessitating considerations around anonymity in the context and mode of data collection [25–27]. The perceived risks of asking about sensitive topics, such as distress, disclosure, and non-response rates, can sometimes overshadow the potential societal benefits of this type of research [28]. Research into sensitive topics, while encompassing potential risks, can be of great importance for policy and practice with CYP, whereas neglecting such research may contribute to avoidance and stigma at a societal level [9]. Indeed, there have been questions regarding the extent of impact of asking about sensitive topics; for instance, Langhinrichsen-Rohling et al. [9] encouraged a distinction between temporary distress in relation to completing measures and the unlikely event of lasting psychological harm.

Studies in this area are frequently conducted in schools, both for epidemiological and evaluative purposes, given a growing emphasis on schools as a context for prevention and promotion [29, 30]. From an ethical standpoint, past research has demonstrated additional challenges when engaging CYP in school-based research. In particular, obtaining valid consent in this context is complicated by the way pupils are generally afforded little choice in how they spend their time in school, meaning that research participation can be misconstrued as compulsory [31–34]. Moreover, a reliance on teachers to introduce and guide pupils through the process of completing measures has been noted as potentially problematic, as they are unlikely to be able to facilitate this process as comprehensively as a researcher involved with the project [35]. There is limited understanding of how the school environment may influence data quality, warranting investigation; for instance, completion of measures alongside peers in a classroom may influence responses to such measures, as past research indicates that the social environment can affect responses to sensitive and socially desirable items [36, 37].

At present, there is relevant literature relating to the experience of CYP research participation more generally [38, 39], as well as school-based research engagement [31–35] and mental health measure completion for clinical purposes [40]. However, to our knowledge, there is no prior research exploring CYP’s experiences of completing mental health and wellbeing measures for school-based research (though a recent study explored school-based completion of self-harm measures [41]). As researchers, there is a responsibility to explore and understand how self-report processes are experienced by CYP in mental health research, including in particular contexts, in order to offer appropriate procedures that are ethical, reliable, and valid, and can meet the needs of this group.

### The current study

We set out to explore the way that CYP perceive and experience completing mental health and wellbeing measures, with a focus on completion in a school context. We focused specifically on completion (i.e., directly responding to measures as participants in a research project) to capture perceptions and experiences of the full experience of engaging with this aspect of research. We have sought to centralise the CYP voice in this study by focusing explicitly on CYP’s perceptions and experiences and by co-authoring the study with a young person (HM, the fifth author; note that HM was independent of and older than the participants in the current study). HM is an expert by experience, having acted as an advisor for the National Health Service (NHS)’s mental health services as well as mental health charities throughout their adolescence and young adulthood, and so was well suited to the aim of the current study.

## Methods

### Design

We adopted an exploratory qualitative design, focusing on interview and focus group data pertaining to the completion of an integrated measurement framework including quantitative mental health and wellbeing measures. This qualitative data was gathered as part of the piloting processes for two school-based projects, each of which had distinct but similarly focused measurement frameworks that were administered through similar procedures (as detailed below). Merging qualitative data across two projects is valuable as it allows for findings that capture more *general* experiences of measure completion, rather than experiences grounded in any single set of measures or context. Our full sample drawn from these projects is 133 CYP aged eight to 16 years. This broad age range allows insight into how researchers can facilitate experiences among this group as a whole, rather than within any one age group.

### Research Project 1 (RP1)

#### Project overview

We used data collected during a formative pilot of the Wellbeing Measurement Framework (WMF), an inventory of measures designed to access a range of mental health and wellbeing indices; specific measures are shown in Table 1. The WMF was designed for use in secondary schools taking part in HeadStart, a 5-year, £58.7 million programme set up by The National Lottery Community Fund exploring ways to improve young people’s mental health and wellbeing. Note that piloting was carried out in non-HeadStart schools and so participants here had no engagement with the wider programme.

**Table 1:** Measures completed by participants

Construct	Measure	Measurement framework		
		RP1	RP2 8–11	RP2 11+
Mental wellbeing	Short Warwick-Edinburgh Mental Wellbeing Scale [95]	✓		
Internalising/externalising difficulties, prosocial behaviour	Strengths and Difficulties Questionnaire [96]	✓		
Emotion regulation	Trait Emotional Intelligence Questionnaire—Adolescent Short Form emotion regulation subscale [97]	✓	✓	✓
Perceived stress	Four-item Perceived Stress Scale [98]	✓		
Protective factors	Culturally adapted Student Resilience Survey subscales [73, 99]	✓	✓^a^	✓^a^
Young carer status	Definition of young carer status with a binary yes/no response option	✓		
Positive wellbeing	Huebner life satisfaction scale [100]		✓	✓
Emotional problems	Short moods and feelings questionnaire [101]		✓	✓
Behavioural problems	Me and my feelings [102]		✓	✓
Peer victimisation	KIDSCREEN-52 [103]		✓	✓
Attitudes to help-seeking	Attitudes to help-seeking [104]			✓
Service use	Short client service receipt inventory—service use subscales [105]		✓	✓
Attitudes to mental illness	Attitudes toward mental illness (stigma) questionnaire [49]			✓
Quality of life	Child health utility 9D [106]		✓	✓
Mental health first aid	Mental Health First Aid interventions from Mental Health First Aid Intentions and Behaviours [107]			✓

RP1: Research Project 1 (Wellbeing Measurement Framework); RP2: Research Project 2 (Education for Wellbeing)

^a^ School connection and problem solving subscales only

#### Participants

65 participants aged 10 to 16 years took part in focus groups at eight schools for the piloting of the WMF (five mainstream schools and three specialist schools). Participants volunteered to participate in focus groups after completing the measurement framework for piloting. As these focus groups were part of a formative piloting process, participants took part anonymously and detailed demographic data were not requested.

#### Measure completion process

The WMF (as shown in Table 1) included measures focused on mental health symptoms, wellbeing, stress, and factors associated with positive outcomes (e.g., family support). Each individual measure was presented sequentially, with participants clicking through to the next measure after each one. Measures comprised of more sensitive items were limited in number by prioritising those most important for addressing key research questions and measures that mostly comprised positively phrased items were presented at the beginning and end of the overall measurement framework. As data collection was for research purposes only (rather than as a screening procedure), data was collected confidentially.

Pupils completed the measurement framework in their education settings, in classrooms with computers. At least two weeks prior, pupils and their parents/carers were provided with an information sheet outlining details of the research, the nature of participation, details of data storage, usage and confidentiality, and contact details, along with an opt-out consent form. Immediately prior to completion, pupils were presented with this information in age-appropriate language, including reiterations that participation was voluntary and data would be treated confidentially (including that researchers did not work at their school, and that parents and teachers would not see their answers). Pupils then gave informed assent by ticking a box to proceed. Researchers facilitated the administration of the measurement framework, reiterating key information, guiding online access, and addressing queries; teachers were also present to offer support in many, though not all, cases. Schools were advised to allocate a standard lesson (i.e., 45–60 min) for pupils to complete the measurement framework.

#### Qualitative data collection

Eight focus groups were conducted. In one class per school, the facilitating researchers asked for volunteers to engage in focus groups immediately following completion of the measurement framework. Researchers carried out focus groups in private rooms in participants’ settings, with group size ranging from six to 11. As focus groups were primarily carried out for formative piloting of the WMF, these sessions were not audio recorded; instead, a second researcher took field notes throughout, documenting participants’ comments as closely as possible.

Focus groups enable participants to explore, compare, and contrast their perceptions and experiences with one another, allowing nuanced discussion and clarification [42, 43]. Researchers used a semi-structured topic guide, which facilitated discussion of key topics alongside unanticipated themes [44]. The topic guide (presented in Table 2) included 11 open-ended questions and probes focused on various aspects of completion, namely understanding of items and wording, likes/dislikes of measures and items, perceptions of length and format, Copies of the measurement framework were provided to avoid reliance on recall and to facilitate specificity in comments.

**Table 2:** Interview and focus group questions

Research Project 1: Focus group questions	
1. Do you have any questions that you want to ask us now that you have answered all of the questions? *If yes*, what?	
2. Were there any questions that you did not understand? *If yes*, which? What was difficult to understand about this/these question(s)?	
3. Were there any questions that you think other people your age might find difficult to understand? *If yes*, which? Why?	
4. Were there any questions that you found confusing? *If yes*, which? What was confusing about this/these question(s)?	
5. Were there any questions that you did not like? *If yes*, which? Why?	
6. Were there any words that you found difficult to understand when answering the questions? *If yes*, which?	
7. Were there any words that you think other people your age might find difficult to understand? *If yes*, which? Why?	
8. What do you think of how the questions look and the layout? Do you have any suggestions for how we could improve it? *If yes*, what?	
9. What did you think about how long it took you to answer all of the questions?	
10. Was there anything that you did not like about answering these kinds of questions? *If yes*, what? Why?	
11. Was there anything that you liked about answering these kinds of questions? *If yes*, what? Why?	

Research Project 2: Interview and focus group questions	
1. Can you tell me about what it was like answering these questions?	
2. Was there anything that you liked about doing this? *What/why?*	
3. Was there anything that you did not like about doing this? *What/why?*	
4. Was there anything that you found difficult about doing this? *What/why?*	
5. What did you think about how long it took you to answer all of the questions?	
6. What did you think about answering the questions on the computer instead of on paper?	
7. Was there anything that you would have liked to have been different about the questionnaire? *What/why?*	

Participation in focus groups required opt-in assent from pupils and opt-out consent from their parents/carers. At the beginning of sessions, researchers verbally reminded participants of key information, including an overview of the project and the nature of participation, and reiterated that participation was entirely voluntary. Ethics approval was granted by the main institute’s Research Ethics Committees (Reference number 8097/002).

### Research Project 2 (RP2)

#### Project overview

We used data gathered within a feasibility study for the Education for Wellbeing (EfW) programme, which trialled and evaluated five universal mental health interventions in English primary and secondary schools, commissioned by the Department for Education [45, 46]. Of these five interventions, three aimed to reduce emotional difficulties and two aimed to increase help-seeking intentions.

#### Participants

68 participants aged eight to 15 years (M = 11.88; SD = 2.06) participated in interviews and focus groups across 10 EfW feasibility study schools in South East England. In RP2, 66% (n = 45) of participants were female and 34% (n = 23) were male, while 45% identified themselves as White British.

#### Measure completion process

Measurement frameworks were tailored to assess intended intervention outcomes and mechanisms, and so included a range of mental health indices. The frameworks differed slightly across age groups for RP2, with versions for both primary-aged (8–11 years) and secondary-aged (11+ years) participants (specific measures presented in each version shown in Table 1). In both versions, each individual measure was presented sequentially, with participants clicking through measures one at a time. Measures comprising mostly positively-phrased items were presented at the beginning and end of the framework. As data collection was for research purposes only (rather than as a screening procedure), data was collected confidentially. The measurement framework was administered both before and after intervention delivery to evaluate effectiveness; qualitative data used here focuses on the experiences of pre-intervention completion.

Pupils completed the measurement framework in classrooms with computers (prior to any intervention). At least two weeks prior, pupils and their parents/carers were provided with an information sheet outlining details of the research, the nature of participation, details of data storage, usage, and confidentiality, and contact details, along with an opt-out consent form. Teachers facilitated sessions with instructions for facilitating online access and key information to reiterate to pupils. Pupils were also presented with key information in age-appropriate language immediately prior to completion, including reiterations that participation was voluntary and data would be treated confidentially (including that researchers did not work at their school, and that parents and teachers would not see their answers). Pupils then gave informed assent by ticking a box to proceed. Schools were advised to allocate a standard lesson (i.e., 45–60 min) for completion of the measurement framework.

#### Qualitative data collection

13 interviews and 11 focus groups were conducted. These sessions focused on experiences of completing the measurement framework as well as wider aspects of the project, and so were conducted 2–4 months after pre-intervention completion of measurement frameworks to allow for intervention delivery (but prior to post-intervention completion). Pupils volunteered to participate in interviews and focus groups (e.g., by submitting an expression of interest form provided by teachers). Sessions were carried out by researchers in private rooms within participants’ settings and were audio-recorded and transcribed verbatim, with group sizes ranging from two to five participants.

Both one-to-one interviews and focus groups were conducted. While interviews facilitate detailed exploration of individual perceptions and experiences, focus groups allow participants to explore, compare, and contrast such perspectives with one another [42, 43]. Researchers used a semi-structured topic guide. Most questions focused on participants’ experiences of interventions, as this was the primary focus of qualitative exploration in RP2, but a sub-section of seven questions focused exclusively on experiences of completing the measurement framework, namely likes/dislikes of measures/items and the completion experience, ease/difficulty of completion, perceptions of length and format, and suggestions for improvement (see Table 2). Copies of the measurement framework were provided to avoid reliance on recall, particularly given the time lapse after completion, and to ensure specificity in comments.

For qualitative data collection, information sheets were provided for participants. Participation required opt-in assent from pupils and opt-in consent from parents/carers. At the beginning of sessions, researchers verbally reminded participants of key information, including an overview of the project and the nature of participation, and reiterated that participation was entirely voluntary. Ethics approval was granted by the main institute’s Research Ethics Committees for qualitative data collection for the feasibility study of the EfW programme (Reference number 7963/003).

### Summary of methods

In total, the current study draws on 32 data sources (i.e., interviews and focus groups) with 133 participants aged eight to 16 across RP1 and RP2. A summary of the methods across the two projects is shown in Table 3.

**Table 3:** Summary of methods across projects

	Research Project 1	Research Project 2	Current study
Participants	65 participants across eight schools; (both mainstream and specialist) Aged 10–16 years.	68 participants across 10 schools; Aged 8 to 15 years (M = 11.88; SD = 2.06) 66% female and 34% male; 45% White British.	133 participants aged 8 to 16 years
Measure completion process	Measurement framework focused on mental health symptoms, wellbeing, and factors for positive outcomes; Sensitive questions placed in the middle of the overall framework; Measures administered online; Completed in classroom in school; Facilitated by researchers and, where possible, teachers; Pupils and parents/carers given information with two weeks notice; Key information presented at start of completion; Approx. 25 min completion time for most pupils.	Measurement framework focused on a range of mental health indices; Measures administered online; Completed in classroom in school; Facilitated by teachers; Teachers reiterated key information before completion.	Similar measure completion processes in place across the two projects (measures focused on mental health and related constructs, completed online in a school setting)
Qualitative data collection	Eight focus groups across eight schools; Participants volunteered to take part; Focus groups immediately after measure completion; Copies of measures supplied; Semi-structured topic guide used; No recording taken; field notes documented by second researcher.	13 interviews and 11 focus groups; Participants volunteered to take part; Data collected two to four months after completion; Copies of measures supplied; Semi-structured topic guide and schedules used; Audio recording taken.	Data collected through semi-structured focus groups and interviews, with copies of measures supplied to facilitate reflection

### Analysis

A thematic analysis was conducted to identify group patterns across the data, utilising Braun and Clarke’s six-step approach [47]. An inductive approach was utilised given the exploratory nature of the study, generating themes from the data itself rather than examining data in relation to existing theoretical models. The first three authors (OD, EA and RM) familiarised themselves with the dataset by reading through each of the data sources and then generated initial codes across 60% of the dataset by systematically coding extracts in NVivo (Version 11; [48]). At this stage these three authors reviewed this coding in unison to agree upon an initial set of themes. Next, the remaining 40% of data was analysed by three further authors against this initial set of themes (ES, KB, AM). Finally, the first author (OD) reviewed, refined, and named final themes in consultation with all authors, including checks against the data and discussion with the study’s young advisor (HM).

## Findings

We developed six main themes to capture CYP’s perceptions and experiences: *Reflecting on emotions during completion; the importance of anonymity; understanding what is going to happen; ease of responding to items; intensity of completion; and interacting with the measure format.* Table 4 presents these six themes alongside associated subthemes and illustrative quotes. This section details and explores the main themes, drawing on participants’ quotes to illustrate the particular aspects that they discussed. All themes were observed to include data from both of the two projects included for analysis. To provide an indication of prevalence across the dataset, we have adopted the following system in reporting these findings: “most cases” where a finding is present for 24 or more of the 32 data sources, “many cases” where this is true of 16–23 sources, “some cases” for 8–15 sources, and “a few cases” where a finding is present for less than 8 cases. However, it is worth noting that this refers to data sources, capturing focus groups and interviews, rather than individual participant-level responses, as we were not able to reliably distinguish between individuals within audio recordings for focus groups.

**Table 4:** Overview of main themes and associated subthemes

Themes and subthemes	Description of subtheme and indicative quote
Theme 1: Reflecting on emotions during completion	
Reflecting on emotions	*In many cases* participants commented that completion enabled them to reflect on their emotions and their life, both good (e.g., friends) and more challenging (e.g., emotion difficulties):*“If you needed to stop your life for a second just to think what’s going on in my life, is it healthy, am I feeling alright, how am I going to deal with the responsibilities?”* (RP2)
Offloading emotions	*In many cases* participants described a process of “releasing” their feelings during completion: *“I felt calm when it was completed”* (RP1)
Help-seeking and aftermath	*In a few cases* participants described feeling differently about how they handled something in their life following completion: *“It’s improved my anger […] I need to stop showing my temper, find another way to calm myself down to fix that situation”* (RP2)
Theme 2: The importance of anonymity	
System anonymity	*In some cases* the anonymity of submitting to researchers was seen as valuable: *“they won’t know who it is”* (RP2), but in a few cases there was concern that schools could check responses and some instances where participants thought they would be identified as needing support: *“others will see and might do something about it”* (RP1)
Surrounded by others during completion	*In a few cases* the presence of peers was sometimes considered acceptable, but *in some cases* participants worried others would see their answers: *“it could make you feel exposed a little bit”* (RP2)
Theme 3: Understanding what is going to happen	
Prior understanding of participation	*In a few cases* there was some lack of clarity on aspects of participation, including how long the process would take and how data would be used: *“I didn’t really know where it was all going”* (RP2)
Understanding participation rights	There was some confusion about whether completion was compulsory and whether they could skip items: *“you should say if you don’t want to do it you can leave the room”* (RP1)
Knowledge of purpose of research	Though some were unclear about elements of the research, *in some cases* participants indicated that they valued knowing that the study could help others in the future: *“it was going into somewhere where it could help you know everyone that did have the problems”* (RP2)
Theme 4: Ease of responding to items	
Complexity of mental health focus	*In some cases* the complexity of mental health was considered to give an opportunity for reflection (*“you [wouldn’t] really usually think of those questions”*; RP2), but *in some other cases* participants said this could also make questions difficult to understand and challenging to answer: *“Questions that you didn’t even know the answer to”* (RP2)
Understanding of items/item clarity	*In some cases* some items were seen as difficult to understand, including issues around complex phrasing, temporal specifications (e.g., in the last two weeks…) and the context of questions (i.e., home versus school): *“I had to ask a teacher like, to explain a question”* (RP2)
Ability of answer options to capture response	Participants described mixed perspectives about Likert scales. *In a few cases* participants commented that they facilitated nuance (*“I think it was a good way to answer because it has like a different variety of answers”*; RP2), in *a few cases* they were described as sometimes confusing (*“it’s difficult to know what’s between”*; RP1), and in *a few cases* these options were seen as restrictive (*“if I could write the answers […] I would’ve explained why*; RP2).
Support from others	*In a few cases* participants reported asking others for support, including peers (*“we were discussing it with each other”; RP2)*, and teachers, though teachers weren’t always viewed as knowledgeable about the measurement framework: *“they didn’t even know how to explain it”* (RP2)
Theme 5: Intensity of completion	
Length of measurement framework and time to complete	There were varying perspectives on how acceptable the measurement framework length was, with participants *in some cases* indicating acceptability (*“I think it was the right length; RP2)* and *in some cases* indicating it took too long: *“it went on forever”* (RP1)
Repetition of items across the measurement framework	*In a few cases* participants felt that items across the measurement framework were sometimes repetitive: *“some repeated itself and […] it’s kind of the same content”* (RP2)
Comfort level	*In many cases* participants commented on the sensitivity of questions, which *in some cases* they felt was sometimes *“too personal”* (RP1)
Theme 6: Interacting with the measure format	
Preference for computer format	*In many cases* computer-based completion was described as preferred to paper completion, offering increased security, anonymity, and accessibility: *“it was easy ‘cause like I’m used to doing it on the computer”* (RP2)
Engaging with the visual format	*In a few cases* participants found the visual formatting difficult to navigate, particularly matching Likert options to individual items: *“it was so close together you could make a mistake”* (RP1)

As in the written narrative of themes, findings are presented here using the following system to indicate prevalence across the 32 data sources: “most cases” where a finding is present for 24 or more of the 32 data sources, “many cases” for 16–23 sources, “some cases” for 8–15 sources, and “a few cases” for less than 8 cases

### Reflecting on emotions during completion

In many cases, completion of the measurement framework was seen as an opportunity to “release” feelings and to reflect on one’s emotions, behaviours, and life; for instance, “*you got to like, look back upon like previous actions and what, what made you feel that way”* (RP2) and *“I felt calm when it was completed”* (RP1). Some of the participants in these cases highlighted that they did not typically have time to reflect in this way on a day-to-day basis, explaining *“you actually got a second to think about it”* (RP2) and *“if you needed to stop your life for a second just to think what’s going on in my life, is it healthy, am I feeling alright, how am I going to deal with the responsibilities?”* (RP2). As part of this, in some cases participants also described identifying elements of their lives that they were less happy with, such as difficulties with emotions; for instance, one participant explained they had *“never thought about them [feelings], now I can work on them”* (RP1). In some other cases, participants described taking stock of the positive aspects of their lives: *“I need to change this. But some I don’t need to change. At least you know, okay, my lifestyle’s all right”* (RP2). In a few cases, participants suggested that this might be uncomfortable for CYP who felt that something was difficult or lacking in their life: “*those that don’t have friends might [not] want to think about it”* (RP1).

There were a few cases where participants explained that completing the measures had made them think differently about how to handle an aspect of their life and wellbeing moving forward, such as reaching out to others or re-evaluating their strategies. For instance, one participant reflected*: “it’s improved my anger […] I need to stop showing my temper, find another way to calm myself down to fix that situation”* (RP2), while another explained that *“you can understand how much you actually might need to talk to somebody or something and not keep it inside if that’s what you were doing”* (RP2). In a few cases, participants therefore highlighted the value of providing information and directions for support at the end of the measurement framework.

### The importance of being anonymous

In some cases, participants commented on the degree of anonymity that they perceived in completing the measurement framework, given that their data would be sent to researchers rather than school staff: *“instead of like… answering them to a teacher so they… know […] you had your own code to get on it so no one could like… figure out what you were answering”* (RP2). In these cases, participants discussed feeling reassured by this and reflected that this particular feature gave them the chance to privately share their feelings, which felt different from talking to someone: *“you’re talking to someone but not actually talking to someone […] they get the thing and the feelings and they won’t know who it is”* (RP2). However, in a few cases participants were less certain about the extent to which their responses were anonymous within this system, and wanted to confirm with the researchers that the school could not see their responses or that nobody would check their individual responses, with questions including *“can someone use your password and check your answers?”* (RP1) and *“these [items and responses] just go to you right?”* (RP1). Indeed, in a few cases participants believed that somebody would see their responses and would then help them: *“if you answer that, others will see and might do something about it”* (RP1). Thus, it was suggested by participants that at the end of the measurement framework there should be an option for participants to disclose that there is something they would like to discuss or need support with, or to opt to share their responses with a teacher: *“at the end you should have a box saying if there is anything you want to talk about”* (RP1).

As noted previously, participants completed the measurement frameworks on computers alongside their peers, in sessions facilitated by researchers and/or teachers. While in a few cases participants stated they were comfortable with other people being present, in some cases participants described feeling exposed and worrying that someone else might look at their responses: *“it could make you feel exposed a little bit”* (RP2). Indeed, in a few cases a participant reported instances of this: “*people would look at your screen. Even though the teachers told you not to, people would still be. I saw people behind me look at each other’s screen”* (RP2). Consequently, in a very small number of cases participants said that they might omit information and provide a false response where items related to behaviours seen as culturally or societally unacceptable. For instance, one participant commented in relation to a question about caregiving responsibilities, which featured a definition that included mention of drug and alcohol abuse: *“for example Muslims cannot have alcohol or [drugs], so if we say yes, someone from the same religion might judge you”* (RP1). Participants gave a number of suggestions as to how this issue could be reduced, namely: (a) allowing completion in smaller groups rather than full classes; (b) ensuring that pupils were not sat directly next to one another; (c) providing a more private space in schools to individually complete the measurement framework (e.g., completing on staff room computers); or (d) sharing the web link with pupils so that they could complete it at home.

### Understanding what is going to happen

In some cases participants seemed to value knowing that the overall study might be helpful to others in the future, and felt that they were making a positive contribution in this way: *“it was going into somewhere where it could help you know everyone that did have the problems”* (RP2). However, in a few cases, participants felt that they had not been fully informed about certain details before they completed the measurement framework. In particular, participants in these cases commented that they were unsure how long the process would take (e.g., *“[I would have liked to know] how long it was gonna go on for*”; RP2), or how the data would be used (e.g., *“I didn’t really know where it was all going”*; RP2), and that they felt that they had been given sufficient advance notice that they were taking part (e.g., *“we only got like two weeks, no two days notice”*; RP2) [insert footnote: as clarified in “Method” section, schools were required to send out information two weeks prior to data collection]. In a few cases, participants asked the researchers these questions during the focus groups and interviews because they had not fully understood at the time of completion. While this demonstrates an interest and desire to understand, it also suggests that these participants did not have the level of information that they wanted about the purpose of the research at the time of completion. In a few cases participants also felt that they had been unclear whether or not completing the measurement framework was compulsory and commented that this should be outlined clearly within the information presented at the start of the measurement framework: *“you should say ‘if you don’t want to do it you can leave the room’”* (RP1). They said they had been uncertain about whether or not they had been able to skip specific items if they wanted to (e.g., *“were we allowed to skip questions?”*; RP1), and felt this too should be made clearer: *“in the beginning say they are personal, but you can skip some”* (RP1). Participants also suggested including a response option that allowed them to explicitly state they didn’t want to respond to an item: *“just have a box so people can say ‘I don’t want to answer’”* (RP1).

### Ease of responding to items

There were a number of comments around how the complexity of mental health as a construct played a role in participants’ experiences. In some cases this was viewed positively, whereby participants felt it meant that there was variety across the overall measurement framework (e.g., *“like, different aspects were included of it”*; RP2) and it also gave them the opportunity to think deeply about their feelings and their life (e.g., *“you [wouldn’t] really usually think of those questions”*; RP2). However, in some other cases participants felt that this complexity made items confusing and difficult to respond to: *“I didn’t really understand the question properly”* (RP2). Often in these instances, participants said that they had not previously considered the types of issues and feelings that they were being asked about: *“what if you’ve never experienced these things?”* (RP1). They frequently highlighted this in relation to hypothetical or scenario-based items; for instance, in a stigma measure, participants were asked whether they agreed or disagreed with a series of statements including “a mentally ill person should not be able to vote in an election” in the Attitudes Toward Mental Illness (Stigma) questionnaire [49]; one participant described these items as *“questions that you didn’t even know the answer to”* (RP2). Some items were seen as unclear due to vague wording (e.g., double-barrelled items, ambiguous wording) and unfamiliar words, which made them difficult to understand: *“I had to ask the teacher like, to explain a question”* (RP2). In a few cases participants highlighted that the temporal nature of the measures, where they were asked to reflect on the last month or the last two weeks, was challenging because they had a difficult time looking beyond how they were feeling on that particular day or beyond specific events: *“if something happened [in the last two weeks], do I consider that or the whole month?”* (RP1). In a few cases, participants were also confused about the context of measures, as they were not sure whether they should only reflect on how they felt at school given that this was where they were taking part, which they commented should be clarified to avoid confusion: *“you should be clear whether it is about home or school”* (RP1).

Likert scale response formats were discussed in many cases, but participants were divided in their comments. In a few cases, participants explained that having different response options available gave them choice and the ability to more accurately capture their feelings. One participant reflected: *“it wasn’t like yes, no, maybe. It was like I’m not sure, but I’m kind of sure, so it’ll be like a seven”* (RP2) while another commented: *“I think it was a good way to answer because it has like different variety of answers”* (RP2). However, in a few other cases participants found the options confusing, with comments including that they didn’t understand the distinctions and scope between the anchors for response options (e.g., strongly disagree to strongly agree, never to always; *“it’s difficult to know what’s between”*; RP1), that some had too many response options (e.g., *“sometimes there are too many boxes”*; RP1), and that these changed across the overall measurement framework (given that multiple measures were combined, each with distinct anchor options). In a few cases, participants said that they wanted a space to provide further detail and explain their responses, as they felt that a numbered response format was restrictive and couldn’t capture the subjectivity of these experiences: *“if I could write the answers, it would be… I would’ve explained why”* (RP2).

In a few cases, participants reported drawing on others around them for support during completion, particularly their peers: *“’cause erm we were discussing it with each other anyway, to know what to say if you didn’t know”* (RP2). In these cases there were participants who recalled asking their teacher to explain something, but it was suggested that the teachers were not necessarily equipped to provide support: *“they don’t even know how to explain it us properly”* (RP2).

### Intensity of completion

There were mixed perspectives on the length of the overall measurement framework and the time it took to complete, with participants in focus groups often disagreeing with each other about this feature, across both projects. In some cases participants indicated that this was acceptable, with comments such as *“I think it was the right length”* (RP2) and *“it didn’t take quite long”* (RP1). However in some cases participants commented that it was too long: *“it went on forever”* (RP1) and in a few cases stated that it could be somewhat repetitive: *“some repeated itself and I was like, it’s kind of the same content”* (RP2). In many cases, participants drew attention to the sensitive or personal nature of some of the items, particularly those focused on mental health symptoms: *“I think the questions to do with emotions and feelings, they are a little bit sensitive”* (RP2). There were a few cases where participants said they recognised the necessity of such items: *“I found a lot of the questions you know very personal, but which was a good thing because it’s […] about you so you know, not other people”* (RP2). However, in some cases participants commented that some items were too personal and that there were a large volume of them; for instance, *“they are too personal”* (RP1) and *“it’s a bit private”* (RP1). In a few cases participants presented this as sometimes uncomfortable and intrusive, with comments such as*“I felt kind of annoyed they’re asking like personal things”* (RP2) and *“we might think it is none of your business”* (RP1). In a few cases participants suggested limiting the amount of these types of items: *“I think just less of, like some of the feelings questions [would help with sensitivity]”* (RP2). In a few cases participants also drew attention to the placement of these types of items within the overall measurement framework, highlighting that as the items were mostly in the middle, this became less difficult over time: *“midway through I wanted to stop because it got personal, but I continued and it got better”* (RP1). In a few cases participants explained that while they were not entirely comfortable with the personal items, these didn’t affect the overall experience; for instance, one participant explained that initially they felt *“a little bit sceptical, because some of the questions were a bit sensitive, but […] all in all, I think it was very helpful”* (RP2).

### Interacting with the measure format

Despite concerns in a few cases around being observed by peers when completing the measurement framework on the computer, in many cases participants said they felt that completing the measures on a computer was better than paper versions, for several reasons. A number of these participants believed that this made their responses more secure and more likely to reach researchers rather than getting lost: *“you believe it’s safer because it’s like, whereas on paper, your answers aren’t going to get lost just like that”* (RP2). These participants also felt that this made the process feel generally more anonymous*: “it felt like you were talking to someone but you were like talking to a computer instead”* (RP2) and meant others wouldn’t be able to figure out that their responses belonged to them: *“[computer was better than paper because] some people can recognise your style of writing”* (RP1). These participants also explained that completing the measurement framework on a computer made the overall process feel familiar and accessible (*“it was easy ‘cause like I’m used to doing it on the computer”;* RP2), and that it was quicker and easier than if they were to complete on paper: *“computer is much quicker”* (RP2). However, in a few cases participants found the visual formatting to be confusing in some places, because they could not always tell which response options related to which item and they suggested making sure information was clearly spread out: *“it was so close together you could make a mistake”* (RP1).

## Discussion

We set out to explore the way that CYP perceive and experience completing mental health and wellbeing measures, with a specific focus on completion in a school context, and developed six main themes: *Reflecting on emotions during completion; the importance of anonymity; understanding what is going to happen; ease of responding to items; level of demand; and interacting with the measure format.* Our findings offer a number of implications, both in relation to optimising the experiences of CYP and for obtaining quality data.

### Measure completion provides a space to reflect

Many participants described reflecting on their emotions, with some describing a “release” seemingly indicative of a lessening either of an emotion or associated burden. Exploring negative emotions is considered valuable and is central within most therapeutic approaches [50–53] and although such inspection can encourage rumination and thus prolong negative affect [54, 55], participants did not describe such difficulties. Thus, findings suggest that responding to mental health and wellbeing measures may facilitate positive reflective processes, rather than distress as sometimes feared with sensitive topics. This complements and extends previous indications that responding to such measures may at worst cause temporary distress and is unlikely to induce lasting psychological harm [9]. The structural design of our measurement frameworks may have facilitated this (e.g., placement of measures with more sensitive items in the middle of the overall measurement framework, limited amount of sensitive items). Such considerations may be important in developing measures and integrated measurement frameworks.

Findings highlight researcher responsibilities to CYP after completion. The emotional reflection processes described, and cases where participants reported wanting to make changes to their life, indicate a need to adequately support CYP to make disclosures or seek support after completion (e.g., having pastoral school staff available). Help-seeking research has drawn on the Theory of Planned Behaviour [56] to emphasise the importance of help-seeking intentions for behaviour change, but also highlights barriers including self- and perceived stigma and low help-seeking efficacy among CYP (e.g., see [57, 58]). Here, in both projects the research teams provided teachers with guidance regarding signposting of support following CYP completion of the measurement framework where appropriate. However, as suggested by participants, researchers could also provide such information directly to CYP at the end of a measurement framework and seek to equip teachers to create de-stigmatising classroom environments that encourage help-seeking.

### Facilitating informed participation

Findings offer insight into several issues and misinterpretations that may arise when CYP engage with participant information, which can influence their experience of the participation process. Firstly, we note that some participants felt they had received insufficient information and prior warning, despite effort from researchers to provide detailed information sheets and two weeks’ notice prior to participation. Similarly, some participants believed someone would see their responses and offer support, which is worrying and warrants careful attention. We note that clear reiteration of confidentiality processes and signposting are key in mitigating this specific misunderstanding, including offering reminders at the end of measure completion; participants did also suggest including an option to disclose difficulties and request support, but this would require careful collaboration with schools to ensure requests are consistently followed through. Taken together, the issues noted above highlight scope for misinterpretation of information, indicating that participant information sheets may not be understood, trusted, or read. Alternative approaches such as video information presentation and provision of clear lesson plans for teachers may better aid understanding and reduce scope for misinterpretation.

Concern about the ambiguous nature of “informed consent” in school-based research is well documented [31–34, 59, 60]. Pupil participation in day-to-day classroom activity is generally compulsory, meaning that research engagement becomes “just another piece of schoolwork” imbued with an assumed lack of choice [31–34], perhaps especially when teachers are the ones introducing the research in large-scale studies. By the time of participation, researchers have negotiated access through gatekeepers in positions of control over CYP, namely teachers and parents, meaning that participation becomes “fait accompli” instead of free choice [31, 34, 59, 60]. Although our participants did not directly draw such links, we note that concerns about being able to opt out or skip items may reflect this context. The power dynamic of the classroom could perhaps be overcome by having non-teaching staff (e.g., pastoral staff) facilitate participation, which could reduce expectations that participation is compulsory, and making other activities available to demonstrate capacity for choice.

Indications that teachers were not perceived as knowledgeable or equipped to offer support also indicate issues for CYP in accessing support in understanding their participation. Here, we implemented several changes following piloting, including developing “crib sheets” of frequently asked questions and relevant information, though we note that not all teachers may engage with such materials given wider workload demands. It may also be important to ensure that such guidance clearly explains ethical processes and boundaries alongside more practical information, so that teachers can provide further guidance and reassurance around issues such as confidentiality to reduce misinterpretation. It is possible that the focus on mental health may be a barrier in this particular context, given that teachers do not always feel confident in supporting pupil mental health and wellbeing [61–63]. The presence of pastoral staff may lessen such issues and facilitate access to informed support as needed.

### Confidentiality and privacy

Findings offer insight into confidentiality and privacy concerns among participants in the context of school-based research. At a system level, participants generally felt their responses were confidential and private, reflecting previous indications that self-administered measures (including online measures) are associated with lower social desirability bias given perceived removal from the researcher [25, 64]. Of course, there were exceptions to this, as some did not trust this confidentiality and others thought this would act as a screening procedure, as discussed above. At a more immediate level, findings suggest peers pose a direct privacy concern in a classroom setting, likely intensified by the ongoing connections that participants have with those around them and, for adolescent participants, heightened sensitivity to peer rejection [65, 66]. Findings indicate that environmental context can introduce a source of anxiety and may prompt false or omitted responses. Researchers could work with schools to develop practices minimising such issues; our participants suggested allowing pupils to complete within smaller groups or within spacious seating arrangements to increase privacy. Finally, we note that although some participants suggested completing measurement frameworks at home to facilitate privacy, this reduces the capacity to ensure there is immediately scope for support. Findings also suggest that issues of social desirability may be heightened among particular groups when others are present, as reflected here in some Muslim participants’ concerns about particular items. This reflects previous findings that cultural norms can introduce social desirability bias [67, 68]. Researchers should be aware of such influences in interpreting findings, particularly in the context of diverse and cross-cultural populations and research. Future research could further explore experiences and barriers across specific groups, including among individuals from diverse ethnic and cultural backgrounds, those with mental health difficulties, and those with additional needs and/or disabilities who may face further practical or cognitive barriers in engaging with measures and/or an integrated measurement framework.

### Interpretability and readability of items and response options

Findings highlight barriers in interpreting items, particularly clarity and familiarity, which influence the extent to which participants feel able to respond. Measurement guidance emphasises the importance of interpretability and readability for reliability (e.g., [69, 70]), yet here even commonly used measures (e.g., the Strengths and Difficulties Questionnaire [SDQ]) were not always clearly understood due to features including unfamiliar vocabulary and double-barrelled items (e.g., “I fight a lot. I can make other people do what I want”; SDQ). Though it is advised all measures (even for adults) should match the typical reading comprehension of a 12-year-old [69], readability studies have shown that CYP mental health measures are frequently not age-appropriate [71, 72]. Findings emphasise that measure developers should carefully consider item readability to ensure age-appropriateness. For researchers adopting pre-existing measures, this highlights the need for piloting regardless of how well validated measures are. Where permitted by developers, researchers could adapt and further validate a measure (e.g., see [73]); where not permitted, researchers could explore alternatives like providing definitions of frequently misunderstood words. Furthermore, although quality guidance advises researchers to specify a time period for respondents (e.g., the last month; [69]), participants found this difficult. Research has shown that the richness of one’s episodic thinking improves in adolescence, while younger children may experience difficulty in immersing themselves in past events [74–76]. This may be particularly problematic in reporting mental health and wellbeing, as more emotionally salient events can be easier to remember and re-construct [77, 78]. Taken together with our findings, this could suggest that younger participants could over- or under-report their overall level of symptomatology or wellbeing. Such findings highlight the benefits of age measurement invariance testing when developing and validating CYP measures, as well as methods such as cognitive interviewing to ensure that items effectively target the intended phenomenon [79–83].

In terms of response options, although some participants reported liking the granularity of the Likert scale, others found this restrictive. Indications that some participants did not feel adequately heard within this narrowed response scope raises questions of whether self-report can truly be considered to centralise CYP voice, as is often suggested [8–10]. Such comments also highlight that quantitative measures alone are insufficient in fully capturing the thoughts, feelings, and experiences of CYP. Participants suggested including open-text boxes alongside quantitative scales to allow elaboration if desired. Of course, Likert scales are inherently subjective given variation in the way participants both items and response options [84, 85]; thus, opportunities to qualitatively contextualise responses may complement quantitative results. However, this would produce large volumes of data, which should be given careful consideration and would warrant different ethical and safeguarding considerations with CYP. Alternatively, broader mixed methods designs with a separate qualitative strand would facilitate deeper understanding of these phenomena and a fuller representation of CYP voice.

Findings also offer insight into the measurement features that constitute burden to CYP when completing mental health and wellbeing measures, namely length, repetition, and item sensitivity, and how they feel this could be mitigated. Aside from the ethical duty to minimise burden, such issues may affect data quality; for instance, inclusion of highly similar items within and across measures can reduce respondent precision [86, 87]. Measure developers should seek to identify small groups of key items where possible and minimise over-similarity across items [88]. Within integrated measurement frameworks, researchers should consider how measures compare with one another to avoid repetition [89]. Finally, it is inherently difficult to measure mental health constructs without sensitive items, and this does not necessarily mean such topics should not be explored. However, there is a need to be mindful about the extent and distribution of such items, which appeared meaningful here given participants’ comments that items “got better” as they went through the framework, and to take ethical steps such as signposting.

### Positive perceptions of computer format

Participants’ reported preference for computer-based participation, rather than paper, reflects previous findings from research with adults [90] and is perhaps unsurprising given current levels of digital literacy among CYP. Here, such comparisons were hypothetical as participants only completed computer-based measures; nevertheless, participants highlighted multiple perceived benefits including greater security, anonymity, familiarity, and accessibility. Some existing research indicates benefits in research with CYP; for instance, Rew, Horner, Riesch, and Cauvin [91] reported higher attention in computer-based completion among school-aged children and suggested that this may feel less like a “test” when completed in schools. However, research indicates data quality issues for computer-based completion; Stieger and Reips [92] found that adults engaged in behaviours associated with lowered data quality, such as changing responses or excessive mouse movement. There is also mixed evidence regarding psychometric effects; though much of this is focused on adults, Patalay and colleagues found item-level differences based on completion mode for the SDQ [93], but not for the Me and My School measure [94]. Currently there is little examination of preferences or differing behaviours across completion mode among CYP, and digital advancements and increased digital literacy among recent generations warrants further up-to-date research.

### Summary of recommendations

Participants’ experiences offer a range of implications and practical considerations for researchers collecting self-report data for child and adolescent mental health research, with additional points to consider in school-based research. We have drawn together the various recommendations outlined throughout this discussion for researchers to consider:

- Present key information to participants in an accessible manner (e.g., videos), as the written information sheets typically used may not be fully digested by participants;
- Ensure that information clarifies the purpose of data collection and how data will/will not be used, including explicit clarity on procedures of anonymity and confidentiality;
- Remind participants of the anonymity and/or confidentiality (as appropriate) of their responses at the end of completion along with clear signposting for relevant avenues of support, and encourage schools (or other delivery agent) to facilitate help-seeking after completion;
- Work with schools to take steps to make clear to CYP that their participation is voluntary rather than compulsory (e.g., having non-teaching staff lead sessions and ensuring alternative activities are available);
- Clearly articulate to participants that they are able to skip items that they do not want to respond to and reiterate this throughout;
- Ensure that steps are taken during completion to facilitate privacy, such as completing in smaller groups or more private spaces than in a typical classroom;
- Researchers should seek to pilot measures and integrated measurement frameworks with CYP prior to main project administration, including use of cognitive interviewing in development of new measures;
- Researchers should work closely with CYP to facilitate readability and interpretability within measures and integrated measurement frameworks, which could be further optimised for a CYP population using age measurement invariance testing and cognitive interviewing; where adaptation is not possible, researchers could provide definitions of frequently misunderstood words to facilitate understanding;
- When integrating multiple measures, inspect overlap and fit across the framework to avoid unnecessary repetitiveness and length;
- It may be beneficial to structure a measurement framework so that measures comprising mostly positive items are presented at the beginning and end to facilitate a more emotionally positive experience; indeed, recent evidence indicates mood-mitigation activities such as a doodle page at the end of a measurement framework may be helpful following emotionally sensitive measure completion [41];
- Including a qualitative strand within the overall project may facilitate a deeper understanding of phenomena and ensure prioritisation of CYP voice; and
- Computer-based administration may be preferable to paper completion for research with CYP.

### Strengths and limitations

A key strength of the current study is its focus on how CYP themselves perceive and experience completing mental health measures for school-based research, including the inclusion of a young person as a co-author. This direct insight into the perspective of CYP is valuable as it can contribute to a clearer understanding of how researcher practices may be perceived by participants, including scope for ethical and data quality implications such as misinterpretation of key information. As a result, the study is able to offer clear recommendations for practice informed directly by CYP, making a timely contribution to the literature given increased use of self-report mental health measures in a school context. Of course, we note that our findings apply specifically to completion of mental health and wellbeing measures for research purposes, in an education context. As such, we highlight that our findings may not be transferable to other contexts, such as mental health screening in schools or assessment for mental health services, given differences in factors such as anonymity. Similarly, the focus of a research project may affect results, such as epidemiological versus experimental designs; here, data from Research Project 1 focused on a pilot sample who completed the measurement framework but were not in the main experimental group (i.e., participants in current study were not participants in HeadStart programme), while in Research Project 2 participants were engaged in an intervention linked with the measurement framework. Although the development of crosscutting themes across two projects is a strength, demonstrating that experiences are not necessarily specific to any one framework or project context, we note that we did not directly ask young people about these wider contexts and indeed were not equipped to compare experiences due to imbalances in the volume of data. Further research should be undertaken to explore how CYP experience completing such measures across a range of contexts and research types, including direct comparisons and exploration of other forms of research engagement such as qualitative engagement.

A number of limitations should be noted. Participants volunteered to engage in interviews and focus groups after completing measurement frameworks, perhaps meaning that those with more positive experiences were more likely to participate, thus potentially affecting the representativeness of findings. Limited demographic information has further reduced our ability to assess representativeness or identify differing group perceptions. Finally, as previously outlined, there was a time lapse of two to four months for collecting qualitative data after completion; while copies of the measurement framework were provided to minimise the effects of this, more immediate responses, particularly emotional ones, may have been lost. We also note that although the current study’s use of a broad age range (eight to 16 years) allows insight into this group as a whole, rather than focusing on any one age group, future research could seek to explore experiences in a design that allows direct examination of variation across age groups.

## Conclusions

We set out to explore the way that CYP perceive and experience completing mental health and wellbeing measures, with a specific focus on completion in a school context, and developed six main themes. Our findings provide insight into the ways that CYP experience completing such measures for school-based research and offer several implications for how researchers and schools can best facilitate this process. Firstly, our findings demonstrate that asking CYP about their thoughts and feelings relating to mental health does not appear to cause damage or long-term distress, but instead can be a valuable experience that allows emotional reflection. Our study also shows it is critical that participation information is presented in a way that is understandable and accessible to ensure that consent is truly “informed”, particularly in the context of completion in education settings. In terms of data quality, it is important that the time and effort CYP invest in participating leads to quality research that can generate robust evidence relating to child and adolescent mental health. This necessitates careful consideration of CYP and public involvement in the development and planning of measures and integrated measurement frameworks for use in such evaluations. We recommend that researchers make clear where such processes have been undertaken and to clarify the steps they have taken to ensure that their data collection processes are designed to best suit the needs of CYP.

## Data Availability

The datasets generated and analysed in the current study are not publicly available due to ethical restrictions.

## Abbreviations

CYP: Children and young people
EfW: Education for Wellbeing
NHS: National Health Service
RP1: Research Project 1
RP2: Research Project 2
SDQ: Strengths and Difficulties Questionnaire
WMF: Wellbeing Measurement Framework

## Footnotes

1 Here we use *measure* to refer to a singular research instrument designed to measure one or more underlying constructs, *item* to refer to the individual statements a participant directly responds to within a measure, *response options* to denote the available answers for a participant to select by way of responding to an item, and *measurement framework* to describe an integrated set of multiple measures administered jointly to create a multivariate dataset. During qualitative data collection with participants however we used “question and answer” for familiarity and ease of discussion.
